# Mucoepidermoid carcinoma of the breast, 3 cases report and literature review

**DOI:** 10.1097/MD.0000000000033707

**Published:** 2023-05-05

**Authors:** Xin He, Jia You, Ying Chen, Hao Tang, Jingni Ran, Deyu Guo

**Affiliations:** a Department of Pathology, Guiqian International General Hospital, Guiyang, China; b Guangzhou Jinyu Medical Clinical Laboratory Center, Guangzhou, China.

**Keywords:** breast cancer, *MAML2*, mucoepidermoid carcinoma, pathologic morphology

## Abstract

Mucoepidermoid carcinoma (MEC) is one of the most common malignant tumors in salivary glands, with specific histomorphological and molecular characteristics. MEC occurring in breast is more rare.

**Patient concerns:**

We reported 3 cases of female breast mass, diagnosed as benign nodules by ultrasound.

**Diagnoses:**

The first 2 cases were pathological diagnosed as breast MEC, low grade, and the third case as breast MEC, medium grade.

**Interventions:**

After pathological diagnosis, 3 patients have expanded the scope of breast resection and lymph node dissection, with negative margin and no lymph node metastasis.

**Outcomes:**

In the follow-up observation, the first case was followed up for 24 months, the second case was followed up for 30 months, and the third case was followed up for 12 months. All patients had a good prognosis without evidence of recurrence and metastasis.

**Conclusion:**

Breast MEC is extremely rare and estrogen receptor, progesterone receptor, and human epidermal growth factor receptor-2 negative breast cancer with a good prognosis, which is different from other highly malignant triple-negative breast cancers. reviewed its clinicopathologic morphological characteristics, immunohistochemical markers and molecular characteristics, prognosis and clinical treatment through literature, in order to understanding its clinicopathology and providing reference for clinical precise treatment.

## 1. Introduction

Breast cancer is the most common malignant tumor in women, and its incidence rate is increasing year by year. In March 2020, the latest data on major cancers in China showed that breast cancer has been ranked first among female cancers, with more than 800 new cases added every day.^[1]^ With increasing awareness of breast cancer, more and more rare types of breast cancer have been reported. Because of its similar structure and cellular composition to the salivary gland, the mammary gland happens the same as salivary gland tumors.^[2]^ The new breast carcinoma type of rare and salivary gland tumors was appearance in the fifth edition of WHO classification of breast tumors. This type of breast salivary gland tumor is usually negative for immunohistochemical markers of estrogen receptor (ER), progesterone receptor (PR), and human epidermal growth factor receptor 2 (HER-2), and most of which have low invasive potential and good prognosis.^[3,4]^ Therefore, it is very important to distinguish them from other highly malignant triple negative breast cancers. Among them, mucoepidermoid carcinoma (MEC) of the breast is reported to account for about 0.2% to 0.3% of the incidence rate of breast cancer. In 1979, Patchefsky et al^[5]^ first reported 2 cases of female breast MEC. So far, MEC of the breast has been reported in 28 published English articles including 42 cases. However, some authors believe that there are more actual breast MEC, which may be due to misdiagnosis. Therefore, it is hoped that the clinicopathologic morphological features, immunohistochemical markers and molecular characteristics of the tumor was understood through the 3 breast MECs and literature review, and provided reference for accurate pathological diagnosis and precise clinical treatment, which is of great significance for the prognosis judgment of patients and the design of diagnosis and treatment scheme.

## 2. Cases presentation

### 2.1. Case 1

**Figure 1.**
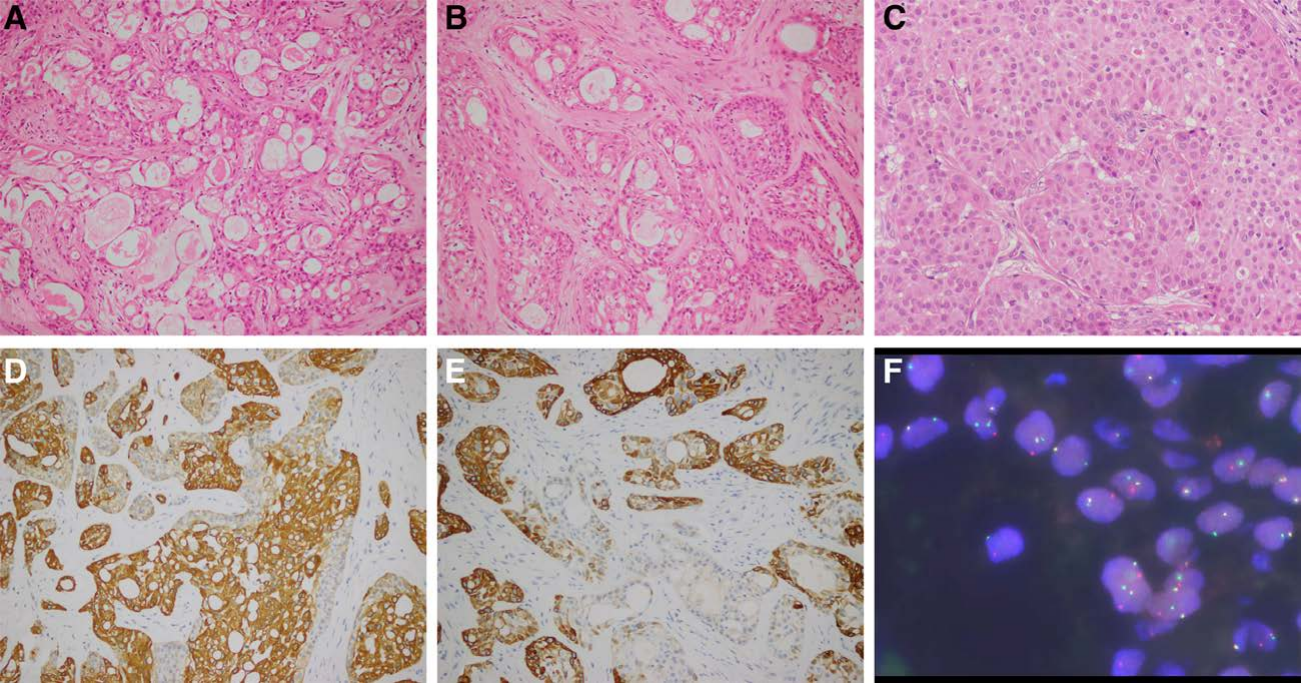
Pathological characteristics of 3 cases breast mucoepidermoid carcinoma. (A) In case 1, the routine pathology of paraffin was mainly cystic area, where mucoid cells were rich in mucus, with a small amount of polygonal cells and rich in eosinophilic cytoplasm (HE × 200). (B) In case 2, the tumor cells were consisted with mucoid cells and polygonal cells arranged alternately, with obvious and small nucleus, nucleoli were seen in polygonal cells and eosinophilic cytoplasm (HE × 200). (C) In case 3, most of the tumor cells were polygonal cells which arranged in solid strip and sheet shape, polygonal cells showed obvious nucleoli, cytoplasm eosinophilic, and nuclear division was easily visible (HE × 400). (D and E) Immunohistochemical results showed that CK7 (D, ×200) was positive in mucoid cells and CK5/6 (E, ×200) positive in polygonal cells. (F) FISH detection showed that there was a broken rearrangement of *MAML2* gene in the case.

A 39-year-old female patient was found a nodule in the outer upper quadrant of the right breast more than 1 year ago. There was no abnormal skin and nipple discharge. Ultrasonography examination showed the nodule was 1.2 cm × 0.8 cm in size with cystic and solid changes, suggestive of a benign tumor. Rapid intraoperative pathological consultation was submitted for breast rotational resection tissue, and there was a pile of gray-white, gray-yellow rotary-cut tissue with a size of 2.0 cm × 1.5 cm × 1.0 cm, medium in texture. Microscopically, most of the tumor cells were mucoid, the cells were large and columnar or round with vacuolar cytoplasm and small and flat nucleus. A small number of tumor cells were mild, polygonal in shape with rich eosinophilic cytoplasm and a round or oval slightly larger nucleus, without defined neurovascular invasion, mitosis and keratinization. The rapid intraoperative pathological consultation was a mucus-rich tumor of the breast, preferring to MEC, which would be confirmed by routine HE and immunohistochemistry staining. Postoperative paraffin routine pathological examination, tumor cells were solid microcystic structure with mucus-rich cytoplasm and flat or irregular nucleus. A small number of polygonal cells with eosinophilic cytoplasm and oval or irregular nucleus was observed around the tumor. There was 1 mitosis per 10 high power fields, no necrosis and nerve invasion were observed. Immunohistochemical results showed that CK7 was positive in mucoid cells, CK5/6 and P63 positive in polygonal cells, and ER, PR and HER-2 negative in all tumor cells. Ki-67 proliferation index was about 5%; periodic acid Schiff staining was positive in mucoid cells; FISH detection showed that there was a broken rearrangement of *MAML2* gene in the tumor cells (Fig. 1).

### 2.2. Case 2

A 37-year-old female patient was found a left breast nodule. Ultrasonography examination showed that the diameter of the nodule was 1.2 cm with well-circumscribed and solid cystic structure, suggestive of an intraductal papilloma. Breast rotational resection tissues were sent for routine pathological examination. In grossly, there was a pile of gray-white, gray-yellow rotary-cut tissue with a size of 2.0 cm × 1.5 cm × 1.0 cm, medium in texture. Microscopically, the tumor cells were consisted with mucoid cells and polygonal cells arranged alternately. The mucoid cells were vacuolate cytoplasm with small and oval nucleus, the polygonal were eosinophilic cytoplasm with round or oval nucleus and obvious nucleolus; No necrosis and nerve invasion were observed. There was 1 mitosis per 10 high power fields (Fig. 1). Immunohistochemical results showed that CK7 was positive in mucoid cells, CK5/6 and P63 positive in polygonal cells, and ER, PR and HER-2 negative in all tumor cells. Ki-67 proliferation index was about 5%; FISH detection showed that there was a broken rearrangement of *MAML2* gene in the tumor cells.

### 2.3. Case 3

A 40-year-old female patient was found a nodule in the lower quadrant of the right breast, which ultrasonography examination showed a diameter of 1.5 cm with well-circumscribed, suggesting intraductal papilloma. Breast rotational resection tissues were sent for routine pathological examination. In grossly, there was a pile of gray-white, gray-yellow rotary-cut tissue with a size of 2.0 cm × 2.0 cm × 1.0 cm, medium in texture. Microscopically, most of the tumor cells were polygonal cells with eosinophilic cytoplasm, round or oval nuclei and evidented nucleolus. The tumor cells were arranged in solid strip and sheet shape. There contained only a very small number of mucoid cells, no necrosis and nerve invasion were observed. There were 4 mitoses per 10 high power fields, and a few lymphocyte infiltration in the interstitium (Fig. 1). Immunohistochemical results showed that CK5/6 and P63 was positive in polygonal cells, CK7, CK8/18, MUC4 positive in mucoid cells, and ER, PR and HER-2 negative in all tumor cells. The proliferation index of Ki-67 was about 20%; FISH detection showed that there was a broken rearrangement of *MAML2* gene in the tumor cells.

## 3. Discussion

MEC is most frequently found in the salivary glands, followed by those in the esophagus, tonsil, thyroid, pleura, lung, thymus, etc.^[6–8]^ It rarely occurs in the breast, with an incidence rate of about 0.2% to 0.3%. Since breast MEC is usually negative for ER, PR, and HER-2, most of them have low invasive potential and good prognosis,^[9]^ so it is very important to differentiate from other highly malignant triple negative breast cancer. Meanwhile, the prognosis of MEC is related to the grading of Elston Ellis scoring system.^[10]^ This grading system is classified as low, medium, and high grade according to the proportion of tumor cystic components, nerve invasion, necrosis, and the number of mitoses per 10 high power fields.^[11]^ Combined with the present cases and relevant English literature, the authors summarized and analyzed the clinical manifestations, pathomorphology and molecular immunohistochemical characteristics, pathological differential diagnosis, clinical treatment and prognosis of breast MEC as follows.

### 3.1. Clinic features

Since the first case of breast MEC was reported in 1979, 45 cases including 3 cases in this report have been reported. Their clinicopathological characteristics were summarized in Table 1. Breast MEC patients are all female in gender with an average age of 54.8 years. There are no obvious symptoms or a few pain in most of the patients. There were clinical symptoms of occasional single breast mass without pain, nipple discharge and other symptoms in 3 cases of the report. No special medical history and family history were found in these patients. Ultrasonography examination showed that the mass showed cystic and solid changes with well-circumscribed, approving for benign tumor.

**Table 1:** Summary of reported cases of breast mucoepidermoid carcinoma from 1979 to 2023.

No	Literature	Publication year	Age (yr)	Size (cm)	Follow-up treatment	Grade	LN metastasis	Distant metastasis	Follow-up care (mo)	Status	*MAML2* rearrangement
1	Present literature	2023	39	1.2	NS	LG	0/NS	NO	24	Alive	YES
2			37	1.2	NS	LG	0/NS	NO	30	Alive	YES
3			40	1.5	NS	IG	0/NS	NO	12	Alive	YES
4	Bak et al^[12]^	2022	47	3.2	CH + RA + ET	IG	0/NS	NO	37	Alive	NS
5	Ye et al^[11]^	2020	42	2.6	CH	LG	0/NS	NO	12	Alive	NS
6	Yan et al^[13]^	2020	60	1.9	NS	LG	NS	NO	60	Alive	YES
7	Burghel et al^[9]^	2017	73	NS	NS	LG	0/2	NO	NS	Alive	APC variant
8	Sherwell-Cabello et al^[14]^	2017	86	6.0	NS	LG	0/NS	NO	3	Alive	NS
9	Cheng et al^[15]^	2017	39	1.5	NS	LG	3/18	NO	156	Alive	NS
10			49	1.5	NS	LG	0/17	NO	41	Alive	NS
11			66	1.3	NS	LG	0/6	NO	9	Alive	NS
12			61	3.0	NS	LG	0/3	NO	4	Alive	NS
13	Fujino et al^[16]^	2016	71	1.7	NS	IG	0/NS	NO	NS	NS	NO
14	Palermo et al^[17]^	2013	80	4.0	NS	HG	0/NS	NO	NS	NS	NS
15	Turk et al^[18]^	2013	40	5.5	CH	NS	1/24	NO	5	Alive	NS
16	Basbug et al^[19]^	2011	69	10	CH + RA	HG	0/12	NO	12	Alive	NS
17	Camelo-Piragua et al^[20]^	2009	49	1.5	CH	IG	1/3	NO	12	Alive	YES
18	Hornychova et al^[2]^	2007	63	1.8	CH + RA	HG	1/18	NO	18	Alive	NS
19			30	8.2	CH + RA	LG	0/NS	NO	60	Alive	NS
20	Horii et al^[21]^	2006	54	2.5	CH	LG	0/NS	NO	36	Alive	NS
21	Gomez-Aracil et al^[22]^	2006	69	7.5	CH	HG	24/28	NO	54	Alive	NS
22	Di Tommaso et al^[23]^	2004	80	0.5	NS	LG	NS	NO	5	Alive	NS
23			29	0.8	NS	LG	NS	NO	90	Alive	NS
24			54	1.5	NS	LG	NS	NO	13	Alive	NS
25			55	0.6	NS	IG	NS	NO	3	Alive	NS
26			36	1.1	NS	HG	NS	NO	18	Alive	NS
27	Tjalma et al^[24]^	2002	58	3.5	RA + ET	LG-HG	1/17	YES	156	Alive	NS
28	Berry et al^[25]^	1998	51	3.5	NS	HG	0/NS	NO	NS	NS	NS
29	Markopoulos et al^[26]^	1998	40	2.0	CH + RA + ET	HG	0/NS	NO	60	Alive	NS
30	Chang et al^[27]^	1998	54	4.5	CH	HG	0/9	NO	48	Alive	NS
31	Luchtrath and Moll^[28]^	1989	60	5.0	CH + RA	HG	12/18	YES	30	DOTD	NS
32	Pettinato et al^[29]^	1989	72	7.0	CH	HG	16/19	YES	10	DOTD	NS
33	Hanna and Kahn^[30]^	1985	51	NS	NS	NS	0/NS	NO	8	Alive	NS
34			31	NS	CH	NS	2/18	NO	14	Alive	NS
35	Hastrup and Sehested^[31]^	1985	59	1.0	CH + RA	HG	0/4	YES	25	DOTD	NS
36	Leong and Williams^[32]^	1985	57	3.5	NS	HG	0/20	YES	7	DOTD	NS
37	Ratanarapee et al^[33]^	1983	27	3.0	CH	HG	6/15	YES	14	DOTD	NS
38	Fisher et al^[34]^	1983	65	2.0	NS	LG	NS	NO	60	Alive	NS
39			71	2.0	NS	LG	0/19	NO	48	Alive	NS
40			57	2.5	NS	LG	0/11	NO	120	Alive	NS
41			49	3.7	NS	LG	0/13	NO	108	Alive	NS
42			60	4.0	NS	LG	NS	NO	48	DOOD	NS
43	Kovi et al^[35]^	1981	46	11	NS	HG	17/19	NS	NS	NS	NS
44	Patchefsky et al^[5]^	1979	66	1.3	NS	LG	0/20	NO	94	DOOD	NS
45			70	5.0	NS	LG	NS	NO	10	Alive	NS

CH = chemotherapy, DOOD = died of other disease, DOTD = died of the disease, ET = endocrine therapy, HG = high grade, IG = intermediate grade, LG = low grade, LN = lymph node, NS = no stated, RA = radiotherapy.

### 3.2. Typical patholomorphologic features

According to reported cases in the English literature, breast MECs range from 0.5 to 11 cm in size, with an average size of 3.2 cm. It is generally a well-circumscribed cystic solid with gray and grayish yellow in section. Histopathologically, it is consisted of polygonal epidermoid cells, intermediate cells and mucoid cells. Mucoid cells are large, with pale cytoplasm and nuclei located at the edge of cells; the intermediate cells are large, with eosinophilic cytoplasm, oval nucleus and small nucleolus; the polygonal epidermoid cells are larger than the intermediate cells, with eosinophilic cytoplasm of no true keratinization^[11]^ and round or oval nucleus. The proportions of the 3 cellular components can vary in different cases. These cells grow solid, cystic or mixed, occasionally papillary or adenoid. Three cases of breast MEC in this paper were strip shaped, gray and yellow, medium in texture, with diameters of 1.2, 1.2, and 1.5 cm respectively. Microscopically, in case 1, the predominant compotents of the tumor were mucoid areas consisted by mucoid cells with mucin-rich cytoplasm and a few areas of polygonal cells with eosinophilic cytoplasm. In case 3, the predominant compotents of the tumor were polygonal cells that were rich in eosinophilic cytoplasm, and the mitoses were easy to be found. In case 2, the areas of mucoid cells were almost similar to that of polygonal cells. The first 2 cases were pathological diagnosed as breast MEC, low grade, and the third case as breast MEC, medium grade.

### 3.3. Immunohistochemical and molecular characteristics

Breast MEC usually presents a typical triple-negative immunophenotype.^[13]^ Most of mucoid cells express low molecular keratin such as CK7, and epidermoid or polygonal cells are positive for high molecular keratin such as CK5/6. However, there were a few cases positive for ER.^[12,15,21,22,30]^ Molecular detection had been detected in four of the 42 breast MECs reported in the previous literature, of which 2 cases reported *MAML2* gene rearrangement, 1 case did not detect *MAML2* gene rearrangement, and 1 case detected APCc. 1703G > A mutation. The immunohistochemical results of 3 cases reported in this paper showed that ER, PR and HER-2 were negative. CK7 was positive in mucoid cells, P63 and CK5/6 positive in epidermal cells or polygonal cells. A broken rearrangement of the *MAML2* (11q21) gene on chromosome 11 were detected in all 3 cases. The exact pathogenesis of MEC in the breast is still unclear. The peers of MEC translocation in the salivary gland are *CRTC1* and *CRTC3*, which belong to the transcription activator family regulated by CREB. The fusion protein of MAML2-CRTC1/3 can activate the downstream target of Notch signal pathway. It is known that abnormal activation of Notch signal pathway can promote the occurrence of breast cancer by down-regulating the expression of E-cadherin, promoting tumor proliferation and angiogenesis, and inhibiting cell apoptosis.

### 3.4. Differential diagnosis

There are 3 kinds of polygonal epidermoid cells, intermediate cells and mucoid cells in breast MEC, which need to be differentiated from tumors with these morphological characteristics. In present case 1, the pathological morphology was mainly mucoid cell region, which should be differentiated from secretory carcinoma. Secretory breast cancer is a rare low grade breast cancer. The tumor cells present a microrod, tubular or solid structure, and produce intracellular and extracellular secretions. Sometimes it is similar to the growth pattern of thyroid follicular epithelium. Immunohistochemical results show that CK7, CK8/18 and EMA are positive, and myoepithelial markers such as P63 are negative, while ER, PR and HER-2 are often negative. The most important molecular feature is *ETV6-NTRK3* gene fusion. In case 2, the pathological morphology contains 3 kinds of cell components, which are typical morphological characteristics of breast MEC. It needs to be differentiated from tumors containing 3 types of cells, such as clear cell papillary sweat adenoma. Clear cell sweat adenoma is a benign skin adnexal tumor with duct differentiation and eccrine differentiation. It can rare occur in the breast and about half locate in the nipple or areola area. The tumor cells are mainly clear and mild morphology. *MAML2* gene rearrangement can be detected in about 50% of patients. There are many overlaps in the histological characteristics and molecular genetic changes between low grade MEC and clear cell sweat adenoma of the breast. Immunohistochemical indicators are helpful to distinguish them. The tumor cells in clear cell sweat adenoma are positive both high molecular keratin and low molecular keratin. The duct and tubular structure lined with cubic cells express SOX-10, but not P63 in clear cell sweat adenoma. In case 3, the pathological morphology is mainly solid polygonal cell area, which needs to be differentiated from invasive ductal carcinoma and squamous cell carcinoma of the breast. Patients with non-special type of invasive breast cancer (invasive ductal cancer) often visit the doctor because of touching the mass, and may be accompanied by nipple discharge, nipple depression, skin ulcer, orange peel and other changes. On the general section, there is a gray and white mass with irregular boundary, showing crab foot infiltrative growth and hard texture. Microscopically, the tumor cells are generally large, atypia, obvious nucleolus and mitoses. The second is breast squamous cell carcinoma. Breast squamous cell carcinoma is generally nodular, irregular in appearance, indistinct in boundary with surrounding tissues, gray and hard in texture, with visible necrosis and infiltrating growth; The tumor cells can be arranged in a nest-like and cystic. The tumor cells are large, eosinophilic cytoplasm , obvious nucleolus, mitoses , and lacking of mucoid cells. Highly differentiated squamous cell carcinoma has keratinized beads, while MEC can have intercellular bridges, but there is no real keratinization. The poorly differentiated squamous cell carcinoma can be confused with MEC with less mucus. The former is obviously atypia, with obvious nucleoli and more mitoses in tumor cells. Immunohistochemical results of ER, PR, and HER-2 can be negative. We can further differentiate diagnosis by mucus staining to show mucoid cells in tumor or FISH detection.

### 3.5. Breast MEC in clinical treatment and prognosis

In the literature, 27 patients with low and medium grade breast MEC had been undergone axillary lymph node dissection, 4 patients showed metastasis with 1 to 3 lymph nodes; Among the 14 high grade patients, 6 patients showed metastasis with 1 to 24 lymph nodes (an average of 12.7). Some patients appeared to be recurrence and distant metastasis. After chemotherapy, radiotherapy and endocrine treatment, 45% of high-grade patients died of this disease. Sherwell-Cabello et al^[14]^ reported that the prognosis of patients with low levels of hormone receptor expression was good. This also indicates that the disease may be hormone-dependent, and endocrine therapy may be an option. Nakano et al^[36]^ reported that patients with *MAML2* gene rearrangement were better prognosis. Three cases of breast MEC patients with *MAML2* gene rearrangement reported in this paper are low and middle grade patients. After pathological diagnosis, 3 patients have been expanded the scope of breast resection and lymph node dissection, with negative margin and no lymph node metastasis. In the follow-up observation, the first case was followed up for 24 months, the second case was followed up for 30 months, and the third case was followed up for 12 months. All patients were a good prognosis without evidence of recurrence and metastasis. Therefore, the choice of surgical method for MEC of the breast should be local excision of the breast tumor and ensure that the margin is negative. Low and middle grade patients can be followed up for observation, but in high grade patients, axillary lymph node dissection should be performed at the same time, and postoperative chemotherapy, radiotherapy and endocrinology treatment should be appropriately added.

In conclusion, breast MEC is a rare breast malignancy. Although the ER, PR and HER-2 of breast MEC are mostly negative, but they are completely different from the clinical treatment and prognosis of triple-negative breast invasive ductal carcinoma, so it is very important for the accurate diagnosis of breast MEC. Due to the few reports of breast MEC cases, it is not completely clear about the biological behavior and long-term prognosis of the tumor, as well as the rational surgical methods and treatment options. We suggest that for the surgical selection of breast MEC is at least tumor resection, and negative margin. The simultaneous axillary lymph node dissection, and adjuvant therapy such as chemotherapy, radiotherapy, endocrine therapy should be appropriately done in high grade breast MEC.

## Acknowledgments

We would like to thank the patient and her family.

## Author contributions

**Conceptualization:** Xin He, Ying Chen, Deyu Guo.

**Data curation:** Xin He, Jia You, Ying Chen, Deyu Guo.

**Formal analysis:** Jia You.

**Investigation:** Xin He, Hao Tang, Deyu Guo.

**Methodology:** Xin He, Hao Tang, Jingni Ran, Deyu Guo.

**Project administration:** Jingni Ran.

**Software:** Jia You.

**Supervision:** Xin He, Hao Tang.

**Validation:** Xin He, Hao Tang.

**Writing – original draft:** Xin He, Ying Chen.

**Writing – review & editing:** Deyu Guo.
